# Aquaporin 1 mediates early responses to osmotic stimuli in endothelial cells via the calmodulin pathway

**DOI:** 10.1002/2211-5463.13020

**Published:** 2020-11-24

**Authors:** Yong Jiang, Chengqi Wang, Rui Ma, Ying Zhao, Xinyue Ma, Jiaxin Wan, Chunxiang Li, Fanghao Chen, Fang Fang, Mingguang Li

**Affiliations:** ^1^ Department of Laboratory Medicine Jilin Medical University China; ^2^ Department of Clinical Medicine Jilin Medical University China; ^3^ Department of Cardiology Jilin Central Hospital China

**Keywords:** aquaporin 1, calmodulin, cell size, osmotic pressure, protein kinase C

## Abstract

The aquaporins (AQPs) are a family of integral membrane proteins which play critical roles in controlling transcellular water movement in various tissues throughout the body. AQP1 helps mediate the cellular response to osmotic stress and tissue water permeability. However, the mechanism by which AQP1 mediates changes in cell volume is not completely clear. Here, we investigated how AQP1 responds to and controls cell volume upon osmotic stimuli during the early phase after the immediate response. Cells overexpressing AQP1 were exposed to hypotonic or hypertonic medium in the presence or absence of staurosporine or W‐7 hydrochloride, and fluorescence imaging was performed at 0, 5, 10, and 15 min later. Osmotic stimuli induced redistribution of AQP1 into the cell membrane, hypotonic stimuli caused cell enlargement, and hypertonic stimuli induced a reduction in cell size, which was blocked by T157A/T239A mutations. Changes in cell size induced by osmotic stimuli were blocked by an antagonist of calmodulin kinase, W‐7 hydrochloride, but not by the PKC inhibitor staurosporine. These results suggest that calmodulin kinase regulates AQP1 activity during the early response to osmotic stimuli.

## Graphical abstract

Upon exposure to osmotic stimuli, AQP1 is phosphorylated and translocates to the cell membrane. After the initial response, the Ca2+/CaMK2 pathway is activated to regulate membrane localization and the activity of AQP1, which leads to a change in cell size and adjustment to the environment. These changes are blocked by the calmodulin antagonist W‐7.

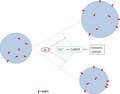

**Abbreviations**
AQP: aquaporin
ERK: extracellular signal‐regulated kinases
HUVEC: human umbilical vein endothelial cells
p38 MAPK: p38‐mitogen‐activated protein kinase
PCR: polymerase chain reaction
PKA: protein kinase A
PKC: protein kinase C

The aquaporins (AQPs) are a family of integral membrane proteins play critical roles in controlling transcellular water movement in various tissues throughout the body [1, 2]. AQP1 has been shown to be strongly expressed in microvessels of the endometrium [3], lung [4], tumors [5, 6, 7], and cirrhotic liver [8]. The growth of xenografted tumor in mice is impaired by AQP1 knockout with reduced tumor angiogenesis and elevated necrosis [9]. AQP1 is strongly expressed in most microvasculature endothelial cells [10, 11] and shown to promote angiogenesis [12, 13]. Cardioplegia, ischemia and hypoxia inhibit cardiac endothelial AQP1 expression [10]. Silencing AQP1 expression results in elevated endothelial permeability of human pulmonary microvascular endothelial cells with downregulation of VE‐cadherin, epithelial Na+ channel (ENaC), and Na‐K ATPase [14].

AQP1 plays an important role in mediating cellular response to osmotic stress and tissue water permeability [15, 16, 17, 18, 19]. Knockout AQP1 gene in mice impairs water osmotic permeability across peritoneal barrier [20], descending vasa recta [21], corneal [15], the pleural surface [22], choroid plexus [16], and maternal‐fetal barrier [19]. The water permeability of cell membrane is decided by the abundance of AQP molecules and the properties of the AQP pore in the cell membrane. The AQP1 pore is thought to be constitutively open and can be reversibly transited to a closed state by membrane tension [23]. The control of AQP1 cellular translocation and membrane permeability is thought to involve protein kinase C and calcium signaling [18]. Hyperosmotic stimuli induce recruitment of AQP1 to plasma via a protein kinase A‐dependent pathway in cultured rat peritoneal mesothelial cells [24, 25]. Direct regulation of AQP gene expression and/or AQP protein degradation can be achieved over a time scale from hours to days [26]. Membrane AQP abundance may be achieved by hormone‐receptor systems [27]. However, the mechanism governing AQP1‐mediated changes in cell volume was not completely clear. This study aims to elucidate how AQP1 responds to and controls cell volume upon osmotic changes within first 15 min (early response) after the immediate response (e.g. over a time scale of seconds).

## Results

### AQP1 mediates tonicity‐induced cell size changes after immediate response

**Fig. 1:**
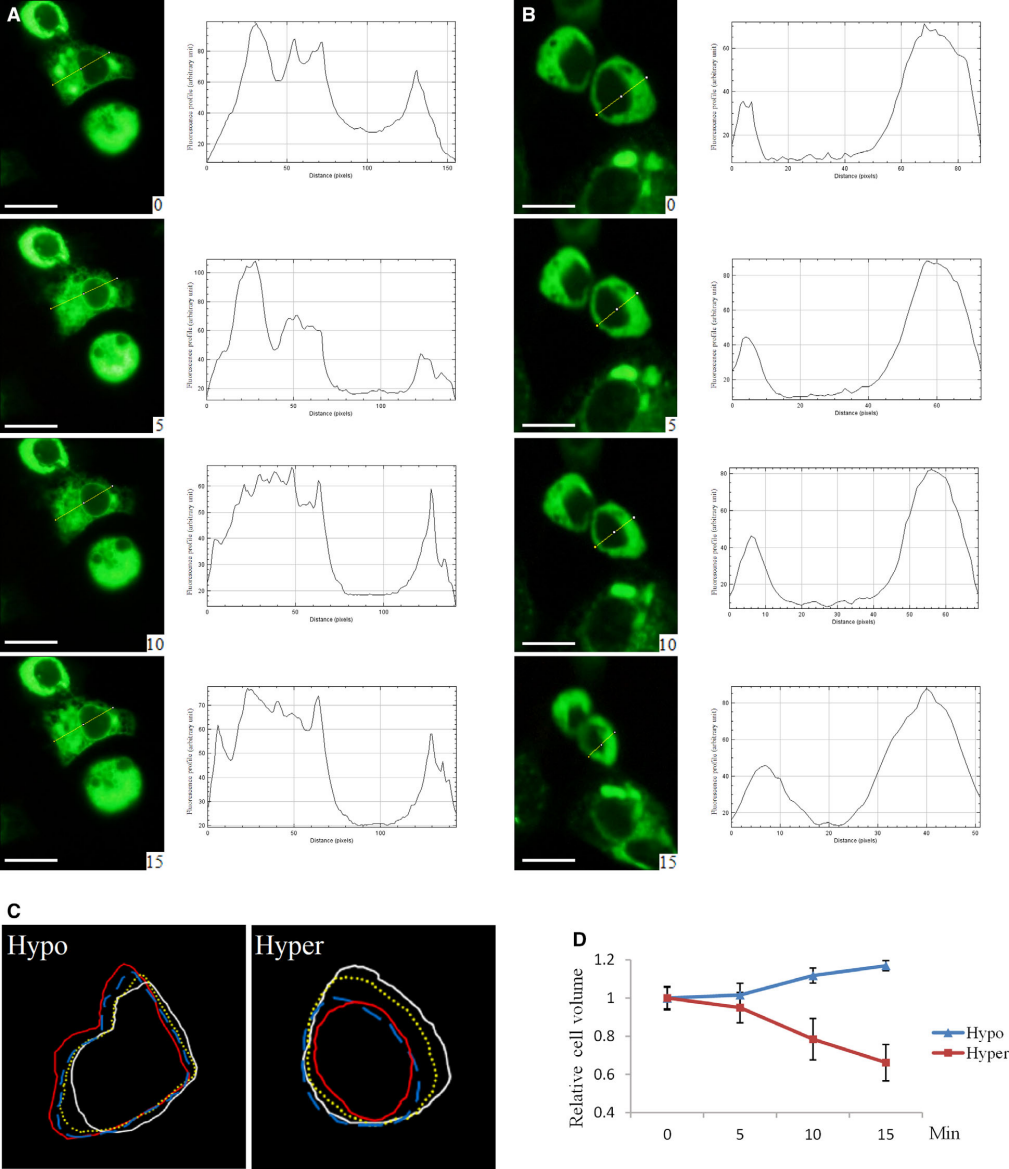
Osmotic stimuli induced redistribution of AQP1 protein and cell size change. HUVEC‐AQP1 cells were transited from isotonic medium into either hypotonic or hypertonic medium and pictured at 0, 5, 10, and 15 min later. The cellular distribution of AQP1 protein in hypotonic (A) and hypertonic (B) media and the change in cell size (C, D) were analyzed using image j software (NIH, Bethesda, MD, USA). For panel C, cell contour of white line was for 0 min, yellow line for 5 min, blue line for 10 min, and red line for 15 min after transiting into hypotonic or hypertonic medium. Data were expressed as mean ± SEM. Scale bar = 50 μm.

As it has been shown that hypotonicity induced AQP1 translocation to cell membrane and cell volume change on a time scale of seconds (immediate response) [18], we looked into the impact of osmotic stimuli on AQP1 cellular location and cell volume on a time scale of minutes (e.g. 5–15 min after exposure to osmotic stimuli). Both hypotonicity (Fig. 1A) and hypertonicity (Fig. 1B) induced AQP1 translocation onto cell membrane at 5–15 min time span. The size of cells was gradually increased in hypotonic medium (Fig. 1A,C,D). On the contrary, hypertonic medium caused the reduction of cell sizes (Fig. 1B–D).

### T157/T239 are required for AQP1 responding to tonic change

**Fig. 2:**
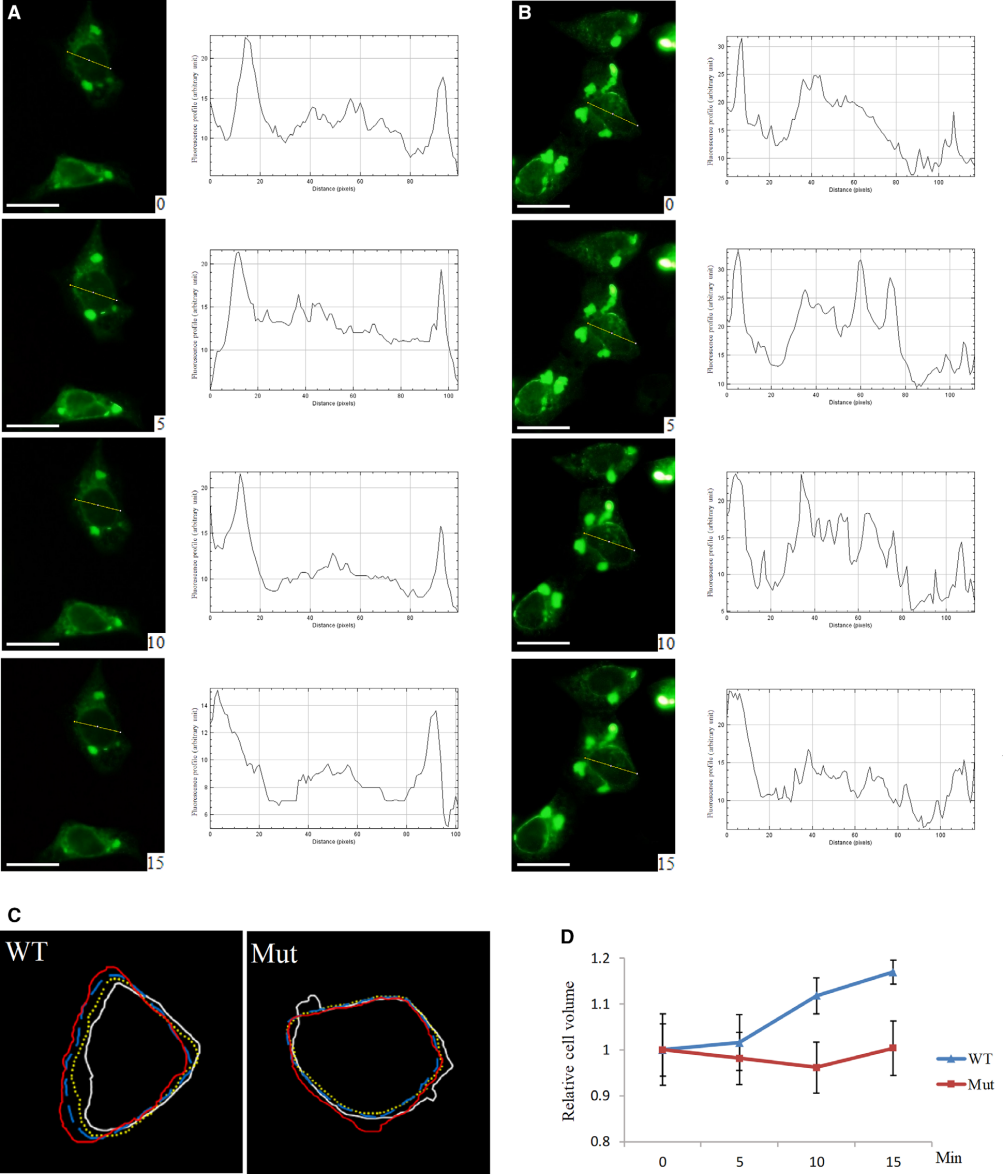
Threonine 157 and 239 were required for AQP1 translocation and activity upon tonic change. Either HUVEC‐AQP1 (A) or HUVEC‐AQP1(T157A/T239A) (B) cells were transited from isotonic medium into hypotonic medium and pictured at 0, 5, 10, and 15 min later. The cellular distribution of AQP1 protein in hypotonic medium and the change in cell size (C, D) were analyzed usingimage jsoftware. For panel C, cell contour of white line was for 0 min, yellow line for 5 min, blue line for 10 min, and red line for 15 min after transition into hypotonic medium. Data were expressed as mean ± SEM. Scale bar = 50 μm.

It has been shown that the immediate AQP1 response to osmotic change was regulated by PKC [27]. We mutated the putative PKC phosphorylation sites of AQP1 threonine 157 (T157) and T239 and assessed the ability of non‐phosphorylation mutant AQP1 responding to tonic change. Mutant AQP1 protein was more evenly distributed throughout cells (Fig. 2B) compared to its wild‐type counterpart (Fig. 2A) under isotonic condition (0 min time point) and did not have obvious membrane translocation seen with wild‐type AQP1 protein upon exposure to osmotic stimuli (Fig 2A,B). Moreover, cells expressing mutant AQP1 did not have significant size change observed with wild‐type AQP1 expressing cells in hypotonic medium (Fig. 2C,D).

### PKC does not regulate AQP1 in early response to osmotic stress

**Fig. 3:**
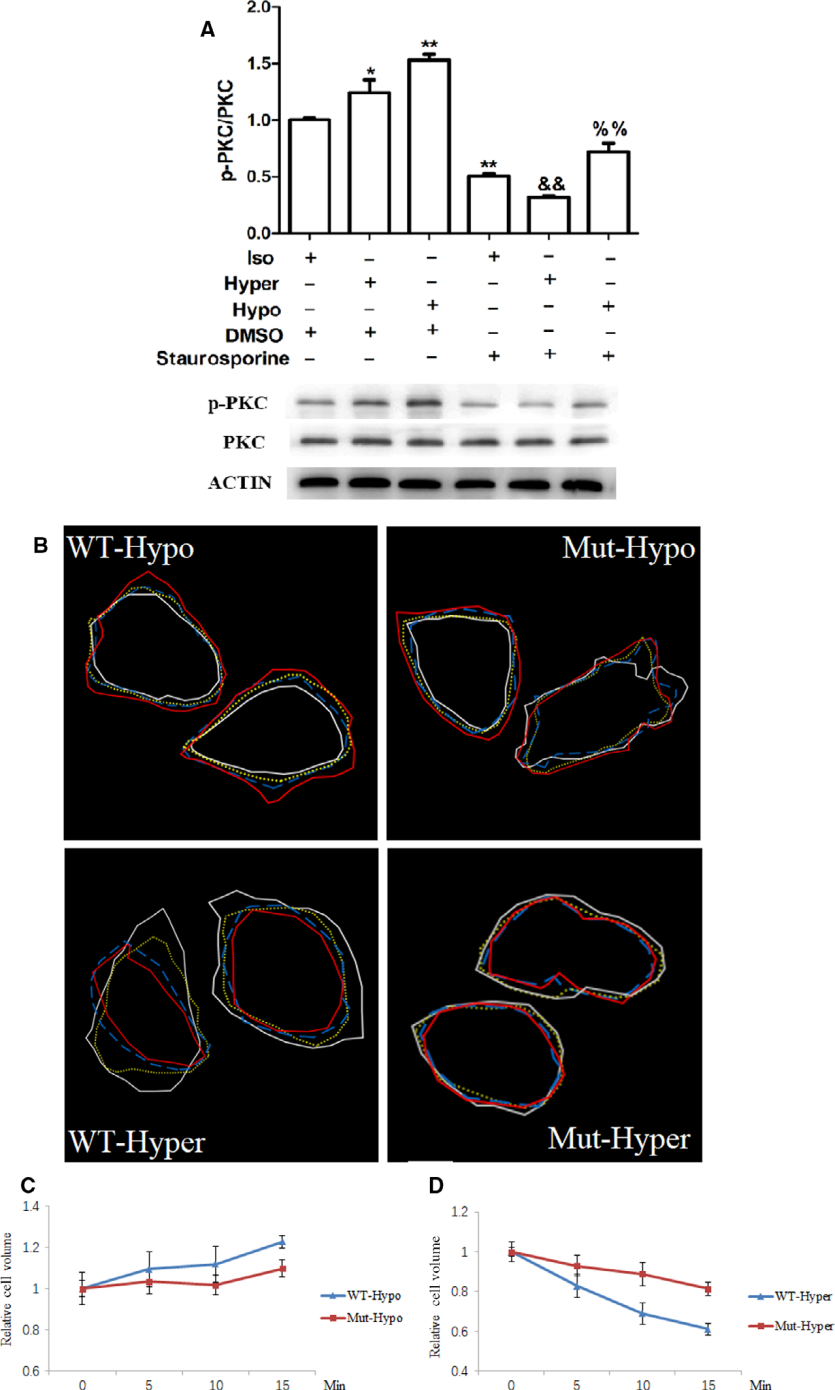
PKC was not responsible for AQP1 early responses to tonic change. (A) HUVECs treated with hypotonic or hypertonic medium with or without staurosporine for 1 min and collected to subject to immunoblotting to detecting the level PKC phosphorylation. (B–D) HUVEC‐AQP1 or HUVEC‐AQP1(T157A/T239A) cells were transited from isotonic medium into hypotonic or hypertonic medium in the presence of staurosporine and pictured at 0, 5, 10, and 15 min later. The changes in cell size were analyzed usingimage jsoftware. For panel B, cell contour of white line was for 0 min, yellow line for 5 min, blue line for 10 min, and red line for 15 min after transiting into hypotonic or hypertonic medium. Data were expressed as mean ± SEM. *:*P* < 0.05, **:*P* < 0.01 compared to isotonic + DMSO; &amp;&amp;:*P* < 0.01 compared to hypertonic + DMSO; %%:*P* < 0.01 compared to hypotonic + DMSO (ANOVA followed by Bonferroni test,*n* = 3–5).

To verify whether PKC activity was required for AQP1 mediated early cellular response to tonic changes after the immediate response, the changes in cell volume after 5–15 min exposure to osmotic stresses in the presence of PKC inhibitor staurosporine were assessed. The level of phosphorylated PKC was increased by hypotonic and hypertonic conditions, which was inhibited by staurosporine (Fig. 3A). However, the inhibition of PKC activation by staurosporine did not reduce the changes in cell size of wild‐type AQP1 expressing cells caused by hypotonicity (Fig. 3B,C) and hypertonicity (Fig. 3B,D). Cells harboring AQP1‐T157A/T239A mutant protein did not change cell size under either hypotonic or hypertonic condition (Fig. 3B–D).

### W‐7 hydrochloride blocks AQP1‐mediated osmotic stress‐caused cellular changes

**Fig. 4:**
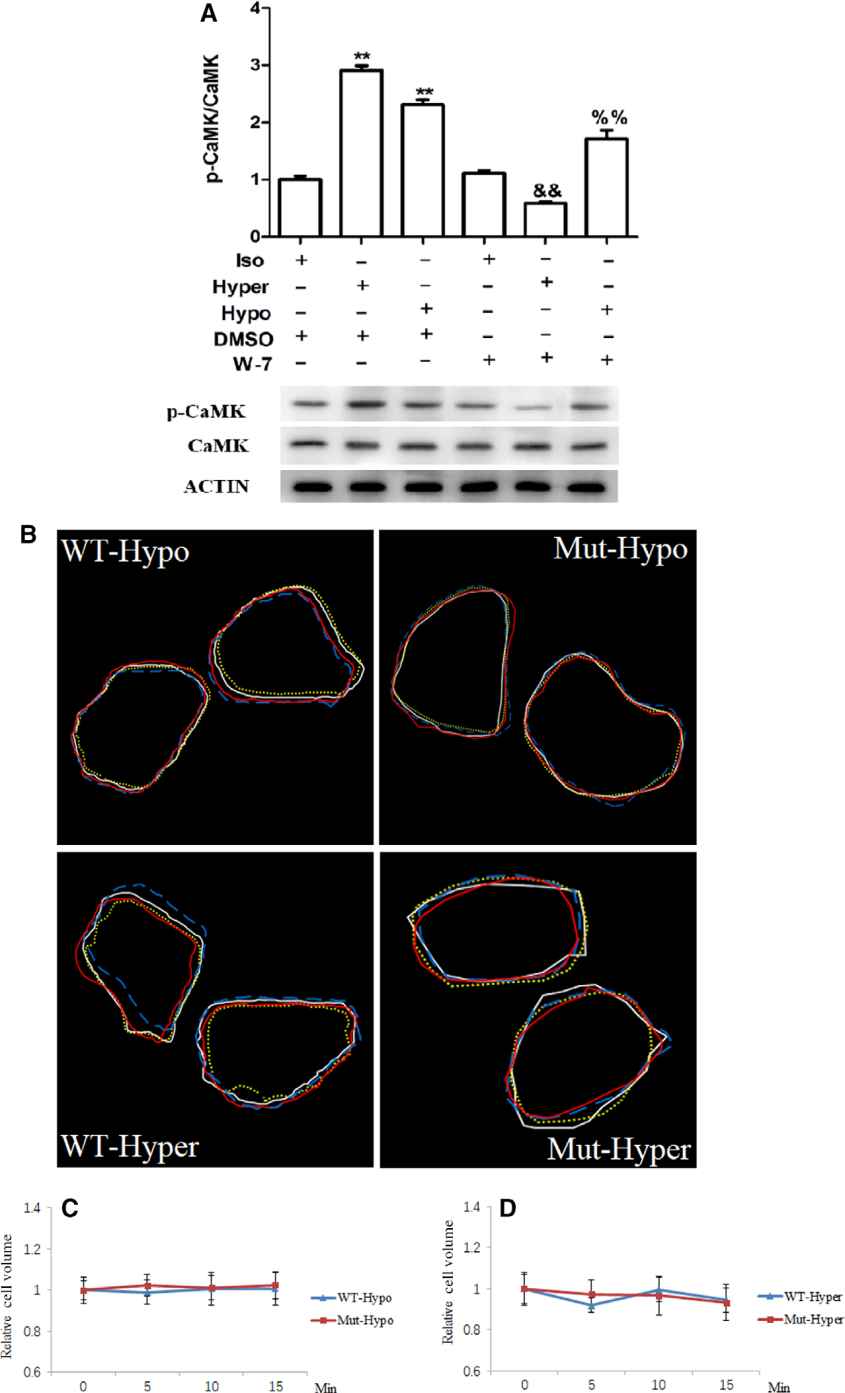
Calcium pathway regulates AQP1 activity after tonic change. (A) HUVECs treated with hypotonic or hypertonic medium with or without W‐7 hydrochloride for 1 min and collected to subject to immunoblotting to detecting the level Calmodulin kinase II phosphorylation. (B–D) HUVEC‐AQP1 or HUVEC‐AQP1(T157A/T239A) cells were transited from isotonic medium into hypotonic or hypertonic medium in the presence of W‐7 hydrochloride and pictured at 0, 5, 10, and 15 min later. The change in cell size were analyzed usingimage jsoftware. For panel B, cell contour of white line was for 0 min, yellow line for 5 min, blue line for 10 min, and red line for 15 min after transiting into hypotonic medium. Data were expressed as mean ± SEM. **:*P* < 0.01 compared to isotonic + DMSO; &amp;&amp;:*P* < 0.01 compared to hypertonic + DMSO; %%:*P* < 0.01 compared to hypotonic + DMSO (ANOVA followed by Bonferroni test,*n* = 3–5).

As Ca^2+^/calmodulin signaling was implicated in the regulation of AQP1 function [18, 28], the impact of calmodulin kinase inhibitor W‐7 hydrochloride (W‐7) on AQP1‐mediated cellular responses to tonic changes was evaluated next. W‐7 substantially inhibited osmotic stress‐induced calmodulin kinase activation (Fig. 4A), which led to diminished changes in cell size of wild‐type AQP1‐expressing cells in both hypotonic (Fig. 4B,C) and hypertonic (Fig. 4B,D) media from 5 to 15 min period.

## Discussion

It is vital to every living organism to maintain water homeostasis. Aquaporins transport water and other small molecules through intra‐subunit pores along osmotic gradients, which plays critical roles in various biological functions, including angiogenesis, cell migration, enabling fluid flow across barrier tissues, maintaining water homeostasis, cellular structure, and cell volume, and supporting metabolic demands among others [29]. Aortic endothelial cells from AQP1‐knockout mice have greatly reduced migratory and vessel formation abilities [9, 12]. The expression level or activity of AQP1 in promoting angiogenesis is regulated by Mef2C and miR‐131a1‐3p [12, 30], estrogen [13], or hypoxia [31]. AQP1 has been shown to regulate the migration of many types of cancer cells [5, 6, 7, 9]. Polarized localization of AQP1 is critical for its roles in cell migration [9, 12].

The translocation of aquaporins to plasma membrane upon tonic changes was shown to be regulated by calcium/calmodulin kinase pathway. Vasopressin increased osmotic water permeability of renal collecting duct by inducing AQP2 translocation onto the apical plasma membrane, which was abrogated by calcium chelator BAPTA, ryanodine receptor antagonist ryanodine, and calmodulin inhibitors W7 and trifluoperazine [32]. Similarly, the accumulation of AQP4 on astrocytes membrane induced by hypothermia was blocked by calcium chelation, calmodulin antagonist trifluoperazine, or transient receptor potential vanilloid 4 antagonist HC‐067047 [33]. Extracellular GTP regulated cortical collecting duct epithelial cell swelling through calcium‐mediated upregulation and membrane localization of AQP5 [34]. Hypoxia induced pulmonary arterial smooth muscle cell migration was mediated by upregulation of AQP1, which was abolished by blockers of calcium channel and siRNA against AQP1 [35]. Moreover, transient membrane translocation of AQP1 might be support by calcium release from intracellular stores but sustained membrane enrichment of AQP1 required influx of extracellular calcium [18]. These results were consistent with the current data that inhibition of calcium‐calmodulin kinase pathway completely blocked osmotic stresses induced membrane translocation of AQP1 and cell volume change at the early response phase (5–15 min after osmotic stimulus exposure).

**Fig. 5:**
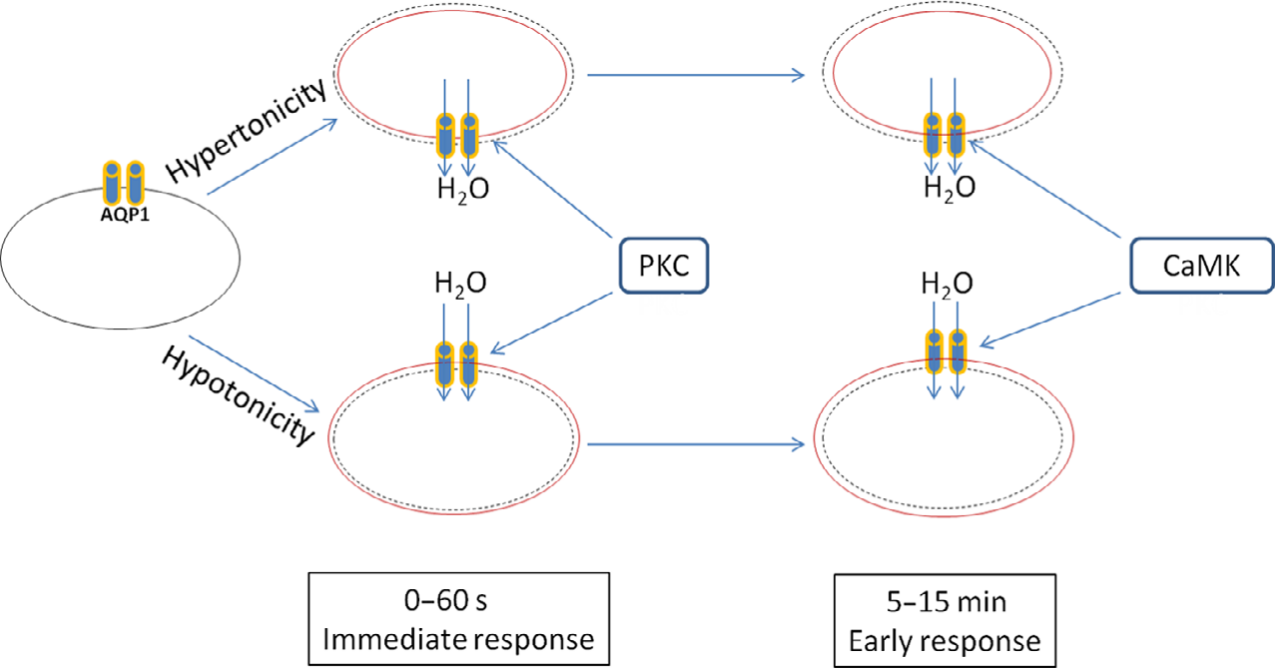
A proposed model to explain how AQP1 was regulated at the immediate response and a early response phases. Dotted shape indicated the original size of the cell while the red line showed the cell size after exposure to osmotic stimuli.

Protein kinases were also shown to play an important role in regulating aquaporin expression, localization, and functions. Vasopressin was shown to stimulate AQP2 phosphorylation and membrane localization in Madin‐Darby canine kidney cells (MDCKCs) via a cAMP/ protein kinase A (PKA) pathway. Moreover, overexpressing a ‘phosphomimic’ mutant AQP2 (AQP2‐S269D) in MDCKCs resulted in constitutive localization at the plasma membrane without any stimulation [36]. However, AQP2 membrane translocation and retention were also stimulated by agonists of vasopressin receptor and prostanoid receptors in a cAMP/PKA independent manner [37]. Hypertonicity induced rapid membrane accumulation of AQP2 in rat kidney collecting duct principal cells in the absence of vasopressin, which was abolished by the inhibition of p38‐mitogen‐activated protein kinase (p38 MAPK) activity [38]. Interestingly, cAMP activated PKA inhibited p38 MAPK activity and in turn decreased AQP2 S261 phosphorylation and polyubiquitination, preventing proteasomal degradation of AQP2 in primary inner medullary collecting duct cells [39]. A biphasic AQP5 response was elicited by cAMP in mouse lung epithelial cells, a decrease in membrane retention and protein abundance of AQP5 was induced by short term cAMP exposure while an increase in AQP5 membrane localization and protein abundance was observed after sustained cAMP treatment [40]. Tonic‐induced rapid AQP4 translocation to membrane was mediated by PKA and calcium/calmodulin activation and only one of the five PKA phosphorylation sites (S276) was required for translocation response [41]. AQP1 translocation to plasma membrane of renal proximal tubules upon acetazolamide treatment was mediated by extracellular signal‐regulated kinases (ERK)/myosin light chain kinase (MLCK) pathway [42]. PKA [25], PKC [18, 27], and calmodulin [18] were shown to regulate osmotic stimuli induced membrane translocation of AQP1. In the current study, AQP1 carrying non‐phosphorylation mutation (T157A/T239A) of two PKC phosphorylation sites [43] did not respond to change in tonicity. However, in the early response phase after the immediate response to osmotic stimuli, inhibition of PKC with staurosporine did not abolish osmotic stimuli induced change in cell shape and cell size, which was in stern contrast with the effect of calmodulin inhibitor W‐7. We postulated that PKC was responsible for the immediate response (within seconds of exposure) of AQP1 to osmotic stimuli, which allowed the quick translocation of AQP1 onto plasma membrane and movement of water molecules. However, once the initial response was over, the activities of AQP1 were regulated by calcium/calmodulin kinase signaling pathway in the early response phase to maintain the cellular adaptation to the environment (Fig. 5).

In conclusion, osmotic stimuli caused membrane translocation of AQP1 protein which mediated hypo‐ and hypertonicity‐induced changes in cell size at the early response period after the initial shock, which was blocked by mutating threonine 157 and 239 into non‐phosphorylatable amino acid alanine. AQP1‐mediated responses to tonic change abrogated by calmodulin kinase inhibitor W‐7 but not by PKC inhibitor staurosporine, indicating that osmotic stimuli might promote AQP1 membrane retention and water permeability at the early response phase (5–15 min after shift into hypotonic or hypertonic medium) through a calcium/CamK regulated but PKC‐independent pathway.

## Materials and methods

### Cell culture

Human umbilical vein endothelial cells (HUVECs) used in this study were obtained from the American Type Culture Collection (Manassas, VA). Cells were cultured at 37 °C and 5% CO_2_ in Endothelial Cell Growth Medium 2 (ECGM2, C‐22010, PromoCell, Heidelberg, Germany). HUVECs used in this study were between passages 15 and 20.

### Construction of plasmids of GFP‐tagged wild‐type or mutated AQP1

Total RNA was isolated from human umbilical vein endothelial cells using a TRIzol reagent (Invitrogen, Shanghai, China). Two micrograms of total RNA was reverse‐transcribed and AQP1 coding sequence was amplified using the following primers: 5′‐CCTAGATCTATGGCCAGCGAGTTCAAGA ‐3′ and 5′‐CTTCAAGCTTGGCTTCATCTCCACCCTG‐3′. The PCR amplification was performed using Phusion^®^ High‐Fidelity DNA Polymerases (New England Biolabs, Ipswich, MA, USA) at 98 °C for 1 min followed by 30 cycles of 98 °C for 10 s, 56 °C for 30 s, 72 °C for 1 min and a final extension at 72 °C for 5 min. The PCR product was gel purified, digested with BglII and HindIII, and ligated into pEGFP‐N1 vector digested by BglII and HindIII. The resultant plasmid was named pEGFP‐N1‐AQP1 and verified by Sanger sequencing.

To obtain AQP1 containing T157A/T239A mutations (two putative PKC sites), pEGFP‐N1‐AQP1 was sequentially amplified with T157A primers (5′‐TGCTGGCTACTGCCGACCGG‐3′ and 5′‐CGGTCGGCAGTAGCCAGCA‐3′) and T239A primers (5′‐GACCTCGCAGACCGCGTG‐3′ and 5′‐CACGCGGTCTGCGAGGTC‐3′). Each round of PCR amplification was performed for 20 cycles with Phusion^®^ High‐Fidelity DNA Polymerases (New England Biolabs) and following cycling conditions: 98 °C for 1 min followed by 20 cycles of 98 °C for 10 s, 56 °C for 30 s, 72 °C for 4 min with a final extension at 72 °C for 10 min. The PCR product was digested with DpnI for 30 min at 37 °C and gel purified before transformation into DH5α cells (New England Biolabs). Mutants were identified by DNA sequencing. The obtained plasmid containing both mutations was named pEGFP‐N1‐AQP1(T157A/T239A).

### Establishment of stable cell line expressing wild‐type or mutant AQP1

Plasmid pEGFP‐N1‐AQP1 and pEGFP‐N1‐AQP1(T157A/T239A) were transfected into HUVECs by Lipofectamine™ 2000 (Invitrogen), respectively. Eight hours later, transfection medium was replaced with fresh ECGM2. Cells were selected with 0.1 mg·mL^−1^ G418 [44] (Invitrogen) and single colonies were isolated and expanded. Stably transfected lines were named as HUVEC‐AQP1 and HUVEC‐AQP1(T157A/T239A).

### Assessment of HUVEC‐AQP1 and HUVEC‐AQP1(T157A/T239A) responses to osmotic stimuli

Both stable cell lines HUVEC‐AQP1 and HUVEC‐AQP1(T157A/T239A) were treated with hypotonic medium or hypertonic medium. Hypotonic medium was made by diluting DEME with deionized water (DMEM : H_2_O = 1 : 2). Hypertonic medium was ECGM2 containing 200 mm sorbitol. To assess the roles of PKC and calmodulin in the distribution and function of AQP1 protein, calmodulin antagonist W‐7 hydrochloride (Cat. No. 0369, Tocris, Minneapolis, MN, USA) and PKC inhibitor staurosporine (Cat. No. 1285, Tocris) were added into culture media while HUVEC‐AQP1 or HUVEC‐AQP1(T157A/T239A) cells were treated by osmotic stimuli. Five minutes, 10 min, and 15 min after osmotic treatment, the changes in AQP1 protein localization in and cell volume of HUVEC‐AQP1 or HUVEC‐AQP1(T157A/T239A) cells were assessed by fluorescence microscope.

### Immunoblot analysis

Sixty seconds after hypotonic or hypertonic medium treatment in the presence or absence of kinase inhibitors, HUVEC cells grown on 6‐cm dishes were washed with ice‐cold phosphate‐buffered saline and resuspended with cell lysis buffer (Beyotime, Shanghai, China) including Halt™ Phosphatase Inhibitor Cocktail (Thermo Fisher, Shanghai, China) and homogenized by 5 passages through a 27‐gauge needle. The homogenate was centrifuged at 13 000 ***g*** for 20 min at 4 °C. The supernatant was collected, and protein concentration was measured using the Bradford protein assay method (Beyotime). Ten micrograms of total protein was resolved on a 10% SDS‐polyacrylamide gel and transferred onto a polyvinylidene fluoride membrane. The membranes were blocked with 5% nonfat milk in PBST for 30 min at room temperature and then incubated with antibodies against phospho‐PKC (ab23513, Abcam, Cambridge, MA), PKC (ab221611, Abcam), phospho‐CaMKII (ab5683, Abcam), CaMKII (ab22609, Abcam) overnight at 4 °C. After washing, the membranes were incubated with anti‐rabbit IgG horseradish peroxidase secondary antibody (Amersham Biosciences, Piscataway, NJ, USA). The immunoreactive bands were visualized using enhanced chemiluminescence method (PerkinElmer Life Sciences, Waltham, MA, USA).

## Conflict of interest

The authors declare that there is no conflict of interest.

## Author contributions

FF and ML designed experiments. YJ, CW, RM, YZ, XM, JW, CL, and FC performed experiments. YJ and ML analysed the results. FF and ML wrote the paper.

## Data Availability

The data will be available from the corresponding author upon reasonable request.
